# Access and Benefit Sharing Under the Nagoya Protocol—*Quo Vadis*? Six Latin American Case Studies Assessing Opportunities and Risk

**DOI:** 10.3389/fphar.2020.00765

**Published:** 2020-06-08

**Authors:** Michael Heinrich, Francesca Scotti, Adolfo Andrade-Cetto, Monica Berger-Gonzalez, Javier Echeverría, Fabio Friso, Felipe Garcia-Cardona, Alan Hesketh, Martin Hitziger, Caroline Maake, Matteo Politi, Carmenza Spadafora, Rita Spadafora

**Affiliations:** ^1^ Research Group ‘Pharmacognosy and Phytotherapy’, UCL School of Pharmacy, University of London, London, United Kingdom; ^2^ Laboratorio de Etnofarmacología, Facultad de Ciencias, Universidad Nacional Autónoma de México, Mexico City, Mexico; ^3^ Centro de Estudios en Salud, Universidad del Valle de Guatemala, Guatemala City, Guatemala; ^4^ Departamento de Ciencias del Ambiente, Facultad de Química y Biología, Universidad de Santiago de Chile, Santiago, Chile; ^5^ Departmento de Investigación, Centro de Rehabilitación de Toxicómanos y de Investigación de Medicinas Tradicionales-Takiwasi, Tarapoto, Peru; ^6^ Instituto de Investigación de Recursos Biológicos, Alexander von Humboldt, Sede Venado del Oro, Bogotá, Colombia; ^7^ Indigena Biodiversity Limited, Gerrards Cross, United Kingdom; ^8^ Section of Epidemiology, Vetsuisse Faculty, University of Zurich, Zurich, Switzerland; ^9^ Institute of Anatomy, University of Zurich, Zurich, Switzerland; ^10^ Department of Pharmacy, University of Chieti-Pescara, Chieti Scalo, Italy; ^11^ Centro de Biología Celular y Molecular de Enfermedades (CBCMe), Instituto de Investigaciones Científicas y Servicios de Alta Tecnología (INDICASAT AIP), Panama City, Panamá; ^12^ Asociación Nacional de Conservación de la Naturaleza (National Association for the Conservation of Nature), Cd. de Panama City, Panamá

**Keywords:** Nagoya Protocol, benefit sharing, traditional medicine, Access and Benefit Sharing (ABS), natural products, biological resources

## Abstract

**Background:**

Global challenges related to access and benefit sharing (ABS) of biological resources have become a key concern in the area of research on herbal medicines, ethnopharmacology, drug discovery, and the development of other high value products for which Intellectual Property protection can be secured. While the Convention on Biological Diversity (CBD, Rio 1992) has been recognized as a huge step forward, the implementation of the Nagoya Protocol (NP) and of new forms of collaboration often remain unresolved, especially in the context of “the fair and equitable sharing of benefits arising from the utilization of genetic resources” (Convention on Biological Diversity, 2011). The vision and the specific implementation of this international treaty vary from country to country, which poses additional challenges.

**Aims:**

Using a case study approach, in this analysis we aim at understanding the specific opportunities and challenges for implementing international collaborations regarding ABS in six Latin American countries—Chile, Colombia, Guatemala, México, Panama, and Peru. Based on that analysis, we provide recommendations for the path ahead regarding international collaborations under ABS agreements in ethnopharmacological research.

**Results and Discussions:**

The implementation of the NP varies in the six countries; and while they are all rich in biodiversity, access and benefit sharing mechanisms differ considerably. There is a need to engage in a consultation process with stakeholders, but this has often come to a halt. Institutional infrastructures to implement national policies are weak, and the level of knowledge about the NP and the CBD within countries remains limited.

**Conclusions:**

Different policies in the six countries result in very diverse strategies and opportunities relating to the equitable use of biodiversity. A long-term strategy is required to facilitate a better understanding of the treaties and the resulting opportunities for a fairer development and implementation of transparent national polices, which currently differ in the six countries. So far, the benefits envisioned by the CBD and the NP remain unfulfilled for all stakeholders involved including local communities.

## Introduction

Ethnopharmacology and, more broadly, natural product research, relies on the access to resources, especially in biodiversity rich regions. Research in this field spans the whole range from, in essence, basic research to studies strictly targeting the commercial development of new products (Heinrich, 2000). It is now governed by a range of international treaties including the Convention on Biological Diversity (CBD) and the Nagoya Protocol (NP). The NP specifically calls for the promotion and safeguarding of “the fair and equitable sharing of benefits arising from the utilization of genetic resources” (Secretariat of the Convention on Biological Diversity, 2011). Therefore, a core focus is now on such benefits which can be commercial or non-commercial. In the current debate, commercial applications are defined widely to include those where there is a long-term potential for a commercial use, but no intention from the researchers to do so (including phytochemical-pharmacological studies). With the nearly global recognition and implementation of the CBD (Rio Convention of 1992, https://www.cbd.int/convention/text/), relationships between nations and territories with regard to the use of biodiversity and its sustainable development have been set on a new base. Grassroot initiatives played a key role in these developments, which aimed to overcome centuries of exploitative relationships often driven by colonial powers. Predating this international treaty are numerous academic and Non-governmental organization (NGO) initiatives including, most importantly, the *Declaration of Belem* of 1988 (http://www.ethnobiology.net/what-we-do/core-programs/global-coalition-2/declaration-of-belem/, see also Posey and Dutfield (1996) that called for a recognition of indigenous rights and for increased support for research on conservation and management programs. Similarly, Tobin (2008) and others highlighted the importance of customary law in protecting traditions. Subsequently, a series of international treaties and protocols [especially the NP in 2014 (https://www.cbd.int/abs/) followed by the Aichi Biodiversity Targets, for the 2011-2020 period (https://www.cbd.int/sp/targets/)] were formulated. Core are the nations’ sovereign rights over the genetic resources found within their national jurisdiction (Ruiz Muller, 2018; Heinrich and Hesketh, 2018), in addition to the necessary involvement in the process, through agreement and Access and Benefit Sharing (ABS), of indigenous people and local communities for granting access based on traditional knowledge related to those resources. The NP also specifically recognizes the traditional knowledge held by indigenous and local communities (Secretariat of the Convention on Biological Diversity, 2011). While such collaborations remain contentious and are seen as a neo-colonial form of exploration by some (Heinrich and Hesketh, 2018), here we want to highlight what opportunities exist based on international agreements and how these can contribute to a more equitable and mutually agreed approach at a national level.

From a different perspective, there have been numerous position papers criticizing exploitative relationships, and this has also been high on the political agenda of some NGOs and other stakeholders (e.g. Dutfield and Suthersanen, 2019 and references therein). While we recognize these problems, in this paper we want to propose specific pragmatic solutions, beneficial to all stakeholders, to develop relationships which result, most importantly, in new opportunities for local communities.

In the field of natural products research, such international efforts are important because plant species hold considerable commercial potential; they provide a huge diversity of chemical complexity, which is still a largely untapped source of novel structural types for pharmaceuticals and consumer products (Heinrich and Prieto-Garcia, 2008). However, many companies have reduced or abandoned their interest in natural products, because of the hurdles in accessing genetic resources (Amirkia and Heinrich, 2015). Yet it is estimated that less than 1% of known plants in the world have been analyzed in detail for their pharmacological activity (Chivian and Bernstein, 2008). Such research is based on fundamental scientific interest and offers multiple opportunities for commercial applications. Very often these aims are poorly understood and differentiated with basic research being at risk due to the “restrictions” imposed by these international treaties (Prathapan et al., 2018). The discussion about ABS is replete with very general statements about challenges and the lack of opportunities to develop partnerships that result in collaborative projects which might yield new high value products for the international market. Scholars have highlighted the implications of these international treaties for international scientific collaboration. Schindel et al. (2015) even claimed “Researchers in industrialized countries reflect back on those open borders as a golden age of research and development.”

Wynberg and Laird (2018) highlighted the “dramatic differences in the pace of policy development and scientific and technological advances” relating to ABS as well as in many other fields. However, the implications of such differences have not been addressed systematically (see Prathapan et al., 2018) and they request *expressis verbis* that “parties to the CBD do more to raise the legal curtain that has fallen between biodiversity scientists and the biodiversity they strive to discover, document, and conserve.” Furthermore, concerns were raised regarding the numerous ambiguities and weak language (in legal terms) in the NP (e.g. Kamau et al., 2010; Kries and Winter, 2015), problems with regards to low priorities for the research in the treaty (e.g. Prathapan et al., 2018) and broadly the “bureaucratic hurdles” resulting from the NP (e.g. http://www.schlich.co.uk/latest_nagoya_protocol.php) and other international agreements. Overall, it is unfortunate that a major factor contributing to the decline in the investigation of plant resources, as potential medicines and consumer products, and for local uses in the countries of origin, has been linked to problems with the implementation of the very international treaties that were intended to encourage it, namely, the CBD and the NP (see *CBD and Commercial Use*). A more fundamental critique is that the current model still maintains in essence a colonialist approach which covers links between countries but does not provide clear guidance on the need for arrangements at a national level regarding equitable access to resources, for example, indigenous knowledge (Wynberg and Laird, 2018). The treaties and the possibilities of developing ABS mechanisms offer an opportunity to overcome such inequalities through national dialogues.

This paper is based on the fundamental premise that such international collaborations are essential and carry opportunities for all those involved, as long as they are based on ethical and legal principles, as outlined in the relevant treaties. Core to any development are economic opportunities. However, major pharmaceutical companies avoid using the NP because of bureaucratic access requirements and legal uncertainties involved in negotiating international agreements (Amirkia and Heinrich, 2015); and countries of origin have been reluctant to work with multinational companies, because of suspicions of “biopiracy” (Ho, 2006). Thus, the two sides, who need to make the NP work, have both withdrawn from the opportunity. The result is that the countries of origin are not gaining the possible benefits from their biodiversity, and a rich source of chemical diversity present in plant species from around the world, is being neglected.

One reason behind this deadlock arises from a mutual lack of understanding between provider countries and users; and also, within governing bodies and their local populations. Companies will have some very specific aims, but many collaborative projects focus primarily on research, which may, but more often does not, result in commercially useful knowledge. From the perspective of the CBD and the NP, this does not matter. Essential is the *potential* commercial application. From the perspective of providers, commonly, the expectations that research will result in commercially successful outcomes are very high. However, all too often this does not reflect reality. Consequently, this discrepancy needs to be assessed in the planning of projects including their non-commercial and potential commercial benefits.

## Aims and Objectives

With a wide range of interpretations of all aspects of these treaties and their implementation, it is essential to understand similarities and differences in core countries which have the potential to benefit from new ABS mechanisms. Here we look at six provider countries in order to compare approaches and solutions for ascertaining access under mutually understood and accepted terms and benefits. We aim at understanding the specific opportunities and challenges in these countries in the context of potential commercial use of biodiversity. While it is not a tool to define how to develop specific agreements, the paper critically compares the approaches of Chile, Colombia, Guatemala, México, Peru, and Panamá, defining what is needed to achieve collaborative partnerships.

## Background

### CBD and Commercial Use

The principle behind the ABS provisions of the CBD and NP is that one country grants access to its genetic resources for utilization by another country; and in return, shares the resulting benefits arising from that utilization. The first issue to understand is the nature of “benefits” and “utilization.”

Art 15.7 of the CBD refers to benefits “arising from the commercial and other utilization of genetic resources” (emphasis added). Thus, it is clear that commercial benefits are contemplated. It follows, then, that if an ABS framework is to be successful, it must attract industry from countries with a strong research base, which is why an understanding of commercial business perspectives is important. Also, Article 11 of the CBD requires countries to adopt measures that “act as incentives for the conservation and sustainable use of components of biological diversity”.

The term “utilization” is defined in the NP as research and development. So, it is important to realize that it is not the genetic resources or plant materials that are being commercialized. That would represent trade of genetic resources as commodities, which is not regulated by NP and CBD. For that reason, the present commentary deals with situations in which genetic resources merely provide the starting point for research. This is important for two reasons: first, the relevance of intellectual property; and secondly, the expectations of benefit.

### Challenges in Cases of Commercial Development

Establishing the level of monetary benefits that is reasonable as part of any benefit-sharing agreement is tricky and often a major point of disagreement between parties. This issue has been a hurdle to successful ABS agreements in the past, often because of misunderstandings and different interpretations of what can be expected realistically.

### The Nagoya Protocol and the Current State of Global ABS Implementation

The NP was adopted by the Conference of the Parties to the Convention on Biological Diversity at its 10th meeting on 29th October 2010 in Nagoya, Japan. It entered into force on October 12th, 2014. By April 2020, the NP had 123 Parties, including the European Union, but excluding several States and territories with notable indigenous populations and often rich biodiversity (Australia, Brazil, Canada, Colombia, Greenland, New Zealand, the Russian Federation, USA, *inter alia*). However, CBD’s near-universal ratification ensures that its more generic provisions for ABS for the sustainable use of biodiversity, such as articles 1, 8 and 15, still apply to all non-Parties to the NP except the USA and the Holy See. Besides manifold national and regional initiatives, an ABS clearing house has also been established within the CBD Secretariat, which assists users and providers to implement ABS provisions (Convention on Biological Diversity, 2019a).

Due to its recent entry into force, aspects of NP implementation that were not solved during initial negotiation are subject to ongoing discussion and definition among Parties. Article 31 establishes an assessment and review mechanism to evaluate the effectiveness of the NP. The first review adopted in Decision NP-3/1 in November 2018 established an indicator framework and reference points against which to measure progress in future assessments (Convention on Biological Diversity, 2018a). By July 2018, 75 Parties had legislative, administrative or policy measures on ABS in place (including many that preceded the adoption of the NP), 44 were in the process of revising or developing new procedures to implement the NP, and 57 had established one or more competent national authorities. Forty-one Parties had legislative, administrative or policy measures in place to implement fair and equitable benefit-sharing on genetic resources held by indigenous people and local communities (IPLCs) and 42 had measures on associated traditional knowledge. Twenty-three parties adopted measures to ensure prior informed consent and involvement of IPLCs, representing 47% of the Parties where IPLCs have the right to grant access to genetic resources. Twenty one parties have taken measures to ensure that traditional knowledge associated with genetic resources is accessed with prior informed consent and involvement of IPLCs under mutually agreed terms, representing 43% of Parties with IPLCs in their country (Convention on Biological Diversity, 2018b). As of April 2020, 1233 internationally recognized certificates of compliance from 22 countries had been published. The vast majority were issued by India (741) or France (233). Latin American countries include Panama (20), Peru (16), Mexico (8), Guyana (5), Uruguay (3), Guatemala and the Dominican Republic (2 each), and Argentina (1) (Convention on Biological Diversity, 2019a).

Article 10 of the NP envisions a global multilateral benefit-sharing mechanism to address cases of genetic resources and traditional knowledge associated with genetic resources that occur in transboundary situations or for which it is not possible to grant or obtain prior informed consent. Decision NP-3/13, adopted at the third meeting of Parties, requests a study to identify: (a) specific cases, if any, that cannot be addressed through the bilateral approach; and (b) if identified, options for addressing these cases, including a possible global multilateral benefit-sharing mechanism, and make a recommendation to the Conference of the Parties serving as the meeting of the Parties to the Nagoya Protocol at its fourth meeting (Convention on Biological Diversity, 2018c). While these issues seem important from a research perspective, Parties consider these to be merely “specific cases”, and not too relevant overall. Some countries indicated that no cases had yet been identified of access to genetic resources or associated traditional knowledge located in the territory of more than one country, and that there was a need to gain more experience on this issue (Convention on Biological Diversity, 2018b).

One of the key challenges recognized in decision NP-3/1 is the implementation of the provisions related to IPLCs. Recommendations include building the capacity of Parties related to IPLCs, and of IPLCs with respect to ABS. This may include national mechanisms for the participation of IPLCs in the NP, coordination and institution building within and among IPLCs (e.g. through community protocols), and support to IPLCs for developing minimum requirements for mutually agreed terms and model contractual clauses (Convention on Biological Diversity, 2018a; Convention on Biological Diversity, 2018b). For the wider legal debate especially relating to indigenous rights (see Tobón-Franco, 2007; Endere and Mariano, 2013; Pacheco, 2015; Pertuzé et al., 2014; Alvarado, 2016; Celi, 2016).

## Approach and Methods

In order to compare the situation in the six countries we used a SWOT analysis, defining the “strengths, weaknesses, opportunities, and threats” resulting from the current state of the implementation of the CBD and the NP including ABS mechanisms. A SWOT analysis is used to assess the current position as a basis for developing a new strategy, in this case for implementing ABS mechanism. It is followed by an internal comparative analysis of the situation in the six countries following the principles of a Delphi process. The strategy is embedded in a project which focuses on the indigenous participation in developing ABS mechanisms in Guatemala. The initial basis was a one-day meeting which included experts from these countries or working with partners in these countries. The initial analysis was then refined by each partner and developed further. The final analysis is based on a consultative round of discussions on various versions of the manuscript.

## Results and Discussion: Case Studies

### Chile

#### The Chilean Flora and Its Uses

The flora of Chile includes over 4500 species (Rodríguez et al., 2018), is highly endemic (ca. 50%) (Cowling et al., 1996) and is increasingly threatened (Olson and Dinerstein, 1998). The inhabitants of Chile have a long history of native plant usage and many indigenous groups continue to use these plants for their subsistence, generating a large body of traditional and local plant knowledge. Díaz-Forestier et al. (2019) identified a total of 995 species of useful vascular plants (23% of Chile’s flora) of which 501 species are described with medicinal uses, 228 with edible uses, 341 used for animal fodder, 300 considered ornamental, 102 used as dyes, 89 for ritual purposes, 75 for timber, and 51 species as a source of fiber. Over 43% of the useful species are endemic to Chile, and 4.7% are threatened (Díaz-Forestier et al., 2019).

#### Implementation of the CBD and the NP

Chile is one of the countries that signed (13/06/1992) and ratified the CBD early on (08/12/1994), but has not yet taken any steps to ratify the NP (https://www.cbd.int/countries/profile/?country=cl). This process is being coordinated by the Comisión Nacional del Medio Ambiente (CONAMA) - now Ministerio de Medio Ambiente y Servicio de Evaluación Ambiental - in its role as the authority responsible for proposing environmental policies to the government and as the national focal point for the CBD.

There are nine indigenous peoples in Chile that represent 9% of its population (1.6 Million). The largest one is Mapuche, followed by the Aymara, the Diaguita, the Lickanantay, and the Quechuas, (International Work Group for Indigenous Affairs; https://www.iwgia.org/en/chile accessed on 24/01/2020). The rural and indigenous communities, together with socio-environmental organizations, signed a declaration asking President V.M. Bachelet (2006 to 2010 and 2014 to 2018) not to ratify the NP on “Access to Genetic Resources and Fair and Equitable Participation in the Benefits arising from its use” in order to avoid a repetition of the internal conflicts resulting from the implementation of another Convention (International Convention for the Protection of New Varieties of Plants) in 2011.

Based on Chilean laws, the implementation of the NP currently requires a consultation with Indigenous Communities and to obtain their free and informed consent. Research projects must give guarantees to the communities with regard to their free access to the results of the research on native and *criollas* seeds, developed in their territory by academic or other entities. Projects must also include short term marketing mechanisms. In this way, the rural and indigenous communities will be able to strengthen and better contribute to the generation of sustainable local economies, and production of healthy and safe food. (Aguilar and Alfaro, 2015).

#### SWOT Analysis

Within the wider framework of biodiversity, there are numerous strengths and opportunities in Chile. However, despite the formal ratification, Chile has not yet developed a structural framework for the overall implementation of the CBD, a necessary basis for the specific formulation of an ABS regime based on previously determined objectives, goals, and priorities. Problems in the context of ABS regulation are a particular concern (**Table 1**). However, the current National Biodiversity Strategy and the future National Biodiversity Action Plan are steps in the right direction (Flores-Mimica and Hervé-Espejo, 2004; Püschel Hoeneisen, 2019). However, this plan does not cover commercial aspects of such a framework.

**Table 1:** SWOT analysis of access and benefit sharing—Case study Chile.

Strengths	Weaknesses
• Since Dec 28, 1994, Chile has been a Party to the CBD, by ratification. **(R)**	• Chile has not signed and is not a party to the NP, nor has it an official competent agency concerned with ABS under the terms of the NP. More broadly, Chile lacks an official inspection body safeguarding ABS. **(R)**
• The Chilean flora includes over 4500 highly endemic species (ca. 50%). **(B)**	• Chile lacks officially recognized “checkpoints” and “checkpoint communiqués” under the terms of Article 17,1(a), (i) of the NP, as well as Internationally Recognized Certificate of Compliance—IRCC under the terms of Article 17 of NP. **(R)**
• Nine hundred ninety-five species of useful vascular plants (23% of Chile's flora), including 501 with medicinal uses, 228 with edible uses, 341 used for animal fodder, 300 with ornamental uses, 102 used as dyes, 89 for ritual purposes, 75 for timber, and 51 species as a source of fiber, are known. **(B)**	• Chile lacks a strong institutional framework for ABS. Access to genetic resource has been nearly exclusively via contracts for academic research purposes, very few for commercial purposes. **(R)**
• Chile has a well-developed Industrial Property Law, also safeguarding and respecting the biological and genetic heritage as well as the national traditional knowledge. **(R/I)**	• There is a great ignorance in the academic sector regarding knowledge of ABS and intellectual property, generating scarce research with commercial opportunities. **(K)**
• Granting of industrial property rights guarantees that this material has been acquired in accordance with the current legal system. **(R/I)**	• A free and informed Indigenous Consultation on the NP (Convention C169 ILO) has not been implemented. **(R)**
	• Despite of a lack of an ABS framework, the Government has already entered into individual ABS agreements, which lack definitions of benefit sharing. (**I/R)**
**Opportunities**	**Threats**
• Chile, being a tricontinental country, presents a great cultural and biological diversity that could be developed. **(B)**	• With most ecozones being fragmented and small, many endemic species are increasingly threatened, as well as traditional knowledge associated with the Chilean flora due to anthropogenic impact including economic activities, e.g., mining, forestry and agriculture industries, and climatic factors, e.g., mega-droughts. **(B)**
• Socio-economic development and preservation of indigenous cultures, in the extreme north and south and on Rapa Nui, with a wealth of traditional knowledge can improve the living conditions in these regions. **(B)**	• The lack of a legal framework on access to genetic resources and associated traditional knowledge could potentially result in major conflicts regarding ABS. **(R)**
• There is no specific ABS framework, but several proposals have been developed (some of them drafted by the agriculture sector), and few ABS agreements have been concluded based on general legal clauses. **(I)**	

Common abbreviations used in all tables: **A**, Academic infrastructure and capacity; **B**, Biodiversity and its use; **G**, General; **I**, Industrial capacity and potential; **K**, Knowledge related to NP and CBD, and **R**, Regulatory framework including the national legal/policy basis.

An inter-ministerial group coordinated by the Ministry of Environment (MMA) was set up in 2010 to study the NP, but the process seems to have been halted since there was no continuity thereof.

##### Legal Implications for Chile of the Non-Ratification of the NP

Current legal challenges include:

- The rights of indigenous and local peoples, especially based on uncertainties, from the ABS point of view, since there is no regulation of access to genetic resources and traditional knowledge,
- scientific research, which is hampered by a lack of legal certainty about access; and;
- as a consequence, a loss of competitiveness as a country with respect to products that could be developed from genetic resources or traditional knowledge associated with them.

The International Union for the Protection of New Varieties of Plants (UPOV) Convention 78 and the Seed Law 19,342 guarantee the rights of breeders of plant varieties, but Chile lacks any specific legislation that safeguards the rights of indigenous peoples and local communities over their genetic heritage and traditional knowledge, which, in the context of the NP, results inajor gaps for implementing the treaty. Similar to other countries (e.g. Guatemala, see below) stakeholders within Chile opposing its ratification believe that it would become an instrument that would only serve transnational corporations and institutions in the global north to generate substantial benefits from the sale of new pharmaceutical, food, beauty or other products. This is a common fear in many biodiversity-rich countries, such as the ones included here. Based on this assessment, they perceive that common goods and ancestral knowledge would be privatized. This view has solidified due to several cases of illegal access that have been reported. A commonly quoted example is that of rapamycin, a drug with antibiotic, immunosuppressor, and anticancer activity, obtained from an endemic bacteria *Streptomyces hygroscopicus* of Rapa Nui (formerly known as Easter Island, Chile) isolated in 1972 from soil samples taken in 1965 (Sehgal et al., 1975). The Chilean Government does not own the genetic resource and does not receive benefits from its commercial exploitation. It seems that association to the indigenous name of the island has prompted local movements into believing its discovery came from traditional knowledge, but this fact has never been assessed (https://issuu.com/moevarua/docs/20_octubre2009; accessed 18/02/2020). This development, which was completed many years prior to the implementation of the treaties, could be seen as an example, of how the CBD and the NP could facilitate equitable benefits, but in Chile, it is more commonly seen as evidence for the lack of benefits of such research for a provider country.

In Chile, there is a particular concern about Article 12.4 (parties “will not restrict as far as possible the customary use and exchange of genetic resources by communities”) regarding the possibility that companies could restrict people’s right to use germplasm (seeds) or patented knowledge for common practices, such as the exchange of seeds, collection, and cultivation of medicinal herbs and the exercise of traditional medicine. This concern stems out of a fear that is politically and socially rooted, as the mentioned article clearly states the contrary. It must be said that “as far as possible” leaves room for exploitation, which would need to be avoided through a specific national regulation aimed at ascertaining that traditional practices do not get hindered due to granted access. Equally, Article 2d of the NP opens the way to modern biotechnology, defining the use of “biological systems, living organisms, or derivatives thereof, to make or modify products or processes for specific use”, contradicting the objectives of biodiversity protection set forth in the introduction to the NP. An obvious solution would be to clearly establish both parties’ requirements and expectations on mutually agreed terms (MTAs) or prior informed consent (PIC). Consequently, in Chile there are major concerns that transgenic agricultural products, for example, from quinoa [*Chenopodium quinoa* Willd), maqui (*Aristotelia chilensis* (Molina) Stuntz], calafate (*Berberis microphylla* G.Forst.) may be the result of the implementation of the NP.

The lack of a legal framework on access to genetic resources and associated traditional knowledge could potentially result in major threats relating to ABS. With the lack of an official inspection body, this could result in a social unrest and economic insecurity, potentially triggering civil conflicts. In general there is a climate of distrust relating to the transmission of traditional knowledge outside the communities, which results in an attitude of “closure” of indigenous people for not seeing benefits from the commercial exploitation of their traditional knowledge.

This situation discourages research collaborations between foreign countries and Chile on Chilean genetic resources, while at the same time still allows unauthorized use of genetic resources and associated traditional knowledge without a fair benefit sharing in exchange for it. Developing a suitable model of implementing the basic principles of the NP would help to resolve these challenges relating to property rights and resolution of conflicts between stakeholders interested in such rights.

### Colombia

#### The Colombian Flora and Its Uses

Colombia is the second country in the world in biodiversity and the first per square kilometer with more than 51,330 species. However, the country’s unknown biodiversity may be almost 50% of its territory. It is the world’s most biodiverse country in birds and orchids, second in plants, amphibians, butterflies, and freshwater fish, third in palms and reptiles and fourth in mammals (SIB Colombia, 2020).

With approximately 25,648 species of flowering plants identified (SIB Colombia, 2020), the country has been implementing a national plant conservation strategy that was welcomed by the CBD in 2002. Of that total, 769 plants are cultivated, almost 798 are under some category of threat and 400 native plants are used for food (Castellanos et al., 2017). Colombia has 1,905,617 indigenous peoples of 87 different ethnolinguistic groups according to its last census in 2018 (https://www.dane.gov.co/files/censo2018/informacion-tecnica/cnpv-2018-presentacion-3ra-entrega.pdf), representing 4.4% of its total population. The great cultural diversity found in Colombia has resulted in a variety of uses of biodiversity. In the case of medicinal plants, 1,656 native species have been recorded in the literature, of which 1,442 are native to the country, and 214 are endemic (Bernal et al., 2011).

#### Implementation of the CBD and the NP

In 1994 Colombia ratified the CBD through Law 165. In 1996, Colombia, together with Bolivia, Ecuador, Peru, and Venezuela, signed the Andean Decision 391 as a “common regime of access to genetic resources” (Stiglitz, 2013; Sarmiento, 2014; Ortiz-Baquero and Solano-Osorio, 2016). The Ministry of Environment was designated as the competent national authority in terms of access to genetic resources. Colombia signed the NP in 2011 and has been working on its ratification (UNDP, 2018), which is pending engagement in prior consultation with indigenous and Afro-descendent communities in the country, a process that was conceptualized as necessary by the Council of State.

However, although not having ratified the NP, the country has strengthened the national ABS framework through Resolution 1348 of 2015 and Decree 1076 of 2015 that clarify which activities require access and compile pre-existing regulations on issues, such as biological collections and species collection permits for non-commercial research purposes. In 2016, a manual was published with the requirements for contracts relating to access to genetic resources and derived products (UNDP, 2018). This manual includes information on the procedures to be followed and the communication strategies that research groups, universities, biotechnology companies, institutions, and organizations need to follow in the dissemination of information about it.

To date, 329 contracts for access to genetic resources have been signed, of which only 11 have been for commercial purposes.

#### SWOT Analysis and Core Future Action Points

Compared to other countries, the regulatory aspects are less of a problem (**Table 2**); instead, the industrial capacity and academic infrastructure are core weaknesses. With a strong institutional framework for ABS in Colombia, there should be multiple opportunities for developing collaborative projects. However, as in other cases, the main challenges relate to accessing biodiversity in an equitable way based on mutual trust and transdisciplinary and international collaboration.

**Table 2:** SWOT analysis of access and benefit sharing—Case study Colombia.

Strengths	Weaknesses
• One of the top megadiverse countries globally. **(B)**	• Access to the genetic resource has been concentrated in contracts for research purposes, very few for commercial purposes. **(A)**
• Colombia has a strong institutional framework for ABS, led by the Ministry of Environment. It has achieved important results especially in the granting of contracts for research purposes, especially with the academy. **(R)**	• Although the country has made significant efforts in regulating access to genetic resources, with an important institutional framework, it still has great weaknesses in access to the biological resources with access being in the hands of regional environmental authorities, institutionally weak to process this type of permits. **(R)**
• The country has important policy documents, CONPES 3697 of 2011 and CONPES 3934 of 2018, which have allowed defining institutional, legal, and economic actions and goals promoting the sustainable use of biodiversity through access to genetic resources. **(R)**	• Despite of a sound academic infrastructure, there are great weaknesses in the academic sector regarding knowledge of ABS and intellectual property, resulting in only very few investigations with commercial opportunities. **(A)**
• The ‘bioeconomy' is an important axis of its development (Law 1955 of 2019 of the National Development Plan). It has prioritized actions in research, development, and innovation, and committed important financial resources. **(R)**	• The Colombian private sector is conservative and has been risk-averse in investing in initiatives to use genetic resources. There is still distrust between academia and industry that prevents capitalizing on important initiatives for the development of new products. **(I)**
**Opportunities**	**Threats**
• Building further collaboration with foreign partners could be based on the great biodiversity linked with a relatively active research program relevant to the sustainable use of biodiversity. **(A/B)**	• There is still a great distrust of local communities regarding access to genetic resources and negotiation with the private sector. This hinders development in many regions of the country with a large presence of ethnic groups. **(I)**
• There is a great interest from international biotech companies, in investing in Colombia, given the wide range of existing biodiversity, with regions with great possibilities for doing business. **(I)**	• There is still a very high expectation regarding royalties resulting from access contracts for commercial purposes, making collaborations commercially unattractive and discouraging companies from investing in such initiatives. **(I)**
• The country has been diversifying its export offer; sectors with great opportunities such as food, cosmetic, and pharmaceutical are emerging rapidly. **(I)**	
• Stakeholders among the local industry could be interested in the use of biodiversity. **(I)**	

The coming years will be marked by actions that seek to concentrate efforts in regions with greater opportunities for the “bioeconomy.” This will lead to further development of bioproducts for different economic sectors, especially for the agricultural, cosmetic, and food sectors. It is generally expected that in the next years the demand for contracts for access to genetic resources for commercial purposes will grow. The Ministry of Environment hopes to reduce the time to grant such contracts and also facilitate access to the biological resource in the regions.

### Guatemala

#### The Guatemalan Flora and Its Uses

Guatemala is one of the 19 megadiverse countries who together host 70% of Earth’s biodiversity. The broad diversity is mostly due to the topographic variations that occur within the country’s borders, producing a complex variety of climatic conditions and, subsequently, ecosystems (Byers and Lopez-Selva, 2016). In 2001, World Wide Fund for Nature (WWF) categorized the national area into 14 “ecoregions” (Olson et al., 2001). This complexity contributes to making Guatemala the Central American country with the highest number of endemic species and a long-standing/ancient traditional knowledge related to them. Despite of the initiatives for a Flora of Guatemala which started mid-20^th^ century (Paul C. Standley and Julian A. Steyermark), the systematic recording of biodiversity, as well as the traditional knowledge associated with it, is far from being exhaustive and the Central highlands are generally best known in terms of their biodiversity and its uses/associated opportunities.

The indigenous population, comprising 21 ethno-linguistic groups, represents around half of the country’s population (UNHCR, 2013; CIA, 2019) and has significant ties to the endemic flora and fauna which still holds considerable spiritual, cultural, and economic importance. Around 79% of the indigenous people suffer from poverty with 40% suffering extreme poverty (CIA, 2019). Traditional ways of life are still important, including a strong reliance on local natural resources for daily lives. Within this context, it is clear how the conservation of Guatemala’s biodiversity assumes a greater social and political significance for the country.

Guatemala’s main source of income and employment is agriculture, with a growing cattle sector expanding in the lowlands of the subtropical rainforest (MAGA, 2016). The expansion of monocultures, such as the African palm (*Elaeis guineensis* Jacq.), and other smaller scale cultivated areas, provides more jobs, but unsustainable use of land will potentially irreversibly undermine the survival of the various ecosystems.

Private interests push the boundaries of the collective goods and land, endangering people’s livelihoods, traditional knowledge, and environmental conservation. The political instability and widespread corruption are the reasons behind a generalized sense of mistrust towards authorities, even those put in place to promote and drive conservation.

#### Implementation of the CBD and the NP

Guatemala signed the CBD early and on October 8, 1995, became a party. However, the implementation of the NP has a far more complex history. Guatemala ratified the NP in October 2014 (Decreto 6-2014, 2014). The ABS national focal point (NFP) for Guatemala is CONAP (Consejo Nacional de Areas Protegidas), which is also the relevant Competent National Authority (CNA). CONAP is the authority in terms of CBD, biodiversity regulations and initiatives, creating the Coordination Department for Indigenous Peoples and Civil Society to develop competence in regard to policies concerning indigenous peoples’ rights.

Guatemala still lacks any policy in place to regulate intellectual property rights and limitations, due in part to the civil society hostility in this regard (Risoli, 2019). A law on agricultural ABS regulates intellectual data but still overlooks the issue of equitable benefits. This puts Guatemala behind all the other Central American countries.

In 2014, Guatemala submitted the National Biodiversity Strategy (NBS) and Action Plan for 2012-2022 (https://www.cbd.int/doc/world/gt/gt-nbsap-v2-es.pdf), outlining the principles for biodiversity conservation and sustainable use, and recently submitted online the 6^th^ National report (2019).

In the months following submission, the NBS, the national authority CONAP started a process for the implementation of NP in the country (even involving an UN-funded project) which nevertheless hit a brick wall in 2016, when the NP was temporarily suspended. The suspension came after the decree was flagged as unconstitutional by an indigenous congressman based on recommendation of the *Gran Consejo de Autoridades Ancestrales de los Pueblos Indígenas de Guatemala* (GCAAG—The Grand Council of Ancestral Authorities of the Indigenous Peoples of Guatemala) and indigenous members of the REDSAG (Network for Food and Nutritional Security), supported by a number of NGOs.

The rationale for the suspension was that the NP had been approved in a rush and without following constitutional regulation mandating that all issues affecting indigenous peoples should follow a consultation process (guaranteed by the national signing of the ILO Convention 169 on Indigenous and Tribal Peoples). Apart from the technical motivations, the driving reason is a justifiable fear of exploitation and discrimination of indigenous people and the wider civil society. Based on information in the popular press, a mistaken association between the so-called Monsanto Law (Law on Protection of Obtaining Vegetable Materials, decree 19-2014, regarding possible genetic manipulation and privatization of native seeds) and the approval of the NP was made. The “Monsanto Law” refers to the use of genetically modified organisms and their use and is not relevant in the context of the NP, but it does highlight the well justified fears of exploitation. A major scandal ensued when civil society representatives uncovered an authorization for a million Quetzal loan hidden within the law’s text. As a consequence, a widespread general mistrust and opposition towards governing bodies dealing with legislation of biodiversity (including CONAP) followed, actively preventing the NP implementation. The ongoing territorial dialogues with indigenous peoples that had been taking place towards formulating a national policy on ABS related to traditional knowledge, which were led by CONAP and an *ad hoc* commission of civil representatives, stopped abruptly. However, this group managed to publish a “Proposal for a Policy on Genetic Resources and Biocultural Heritage” (CONAP, 2016) and has continued lobbying to pass a specific law related to this policy, publishing in 2018 a “Proposal for a law for the protection of biological diversity and the biocultural heritage of indigenous groups and local communities of Guatemala” (CONAP, 2018a). So far, this law has not been formally presented to the Congress. In general, there is fear that commercialization of natural resources will lead to diminished access for people to those same resources (either physical or due to price increase, or increase in standardization and regulation of something that is traditionally traded and exchanged freely). The competent authority itself has clearly indicated that the country’s legislation has contradicting regulations that do not guarantee equitable ABS to indigenous groups. For this reason, it has attempted to generate initial experiences with indigenous groups on access to biodiversity by elaborating biocultural inventories that can set a precedent on collective intellectual property (CONAP, 2018b).

Other relevant norms and regulations in place in Guatemala, in regard to biodiversity conservation and use, ABS and genetic engineering, are: Governmental Agreement 220-2011, National Biodiversity Policy (complemented by Decree Law 4/89), Ministerial Agreement 117-95 (1995) on agricultural ABS (regulating ABS for plants genetic resources, mostly for agricultural use) and the National Biosecurity Policy (2015), which provides guidelines in regard to genetic engineering of national genetic resources.

Currently there is no law regulating intellectual property matters, but they are evaluated case by case by the Intellectual Property Registry, belonging to the Ministry of Economy, while the Ministry of Agriculture and Livestock is the national competent authority for the international treaty on Plant Genetic Resources for Food and Agriculture (Muller, 2016).

#### SWOT Analysis and Core Future Action Points

The unclear legal situation has resulted in an ambiguous and unfavorable environment, which hampers the development of new opportunities. Problems in the context of regulating ABS are a particular concern (**Table 3**). As in other countries, limited communication about the principles and implementation of the CBD’s ABS and a mistrust in the authorities in general, as well as in institutions, has resulted in a halt of the NP and a seemingly lack of interest in projects on the sustainable use of Guatemala’s biodiversity. This, alongside a lack of practical tools for legal access to genetic resources, has resulted in commercial projects/endeavors being withdrawn and taken up in other countries with easier or, at least, clearer stance and requirements. Looking ahead, it seems evident that no policy affecting indigenous peoples’ knowledge will be successfully implemented without ample participation and representation processes, which requires a degree of political will going beyond the NFP’s area of influence and into the heart of state policy.

**Table 3:** SWOT analysis of access and benefit sharing—Case study Guatemala.

Strengths	Weaknesses
• The Central American country with the most endemic species and rich in natural resources. **(B)**	• The suspension of the NP created a situation of uncertainty for foreign entities interested in accessing and potentially developing local resources, pushing potential involvement abroad to countries such as Panama with easier routes to access. **(R)**
• One of the countries with the biggest proportion of indigenous population in Latin America with a long-standing tradition of use and reliance on natural resources (conservation). **(G)**	• No framework in place to support the evaluation of who is the owner of TK and GRs. **(R)**
• CONAP's extensive experience of CBD and NP initiatives and established position for the safeguarding of the environment and indigenous rights. **(R)**	• No policy in place to regulate Intellectual Property rights and limitations. **(R)**
• A strong sense of community/belonging/identity around natural resources. **(K)**	• Unrealistic expectations in relation to potential royalties arising from commercialization of products deriving from genetic resources. **(I)**
• The NBSAP, even though published before the suspension of NP, shows a definite intention of the State to engage with such issues. **(R)**	• General mistrust of authorities and a widespread misinformation on the content of the NP manipulated for political purposes, surfing on indigenous' people malcontent. **(K/R)**
	• There is a very weak local industry interested in the use of biodiversity. **(I)**
	• Weak academic infrastructure. **(A)**
**Opportunities**	**Threats**
• There is a long track record of research collaborations with a variety of international partners based on the local expertise and international links. **(A/I)**	• Major concerns about corruption and political instability result in a lack of willingness of international stakeholders to develop links with institutions in Guatemala. **(G)**
• As highlighted by a previous project (Risoli, 2019), despite the external controversial environment, it is possible to engage in ABS implementation initiatives based in local communities. **(R)**	• High levels of poverty. **(G)**
	• Agricultural expansion and unsustainable practices. **(B)**
	• Drug cartels control over land management and development. **(G)**

### México

#### The Mexican Flora and Its Uses

Mexico stands out among the mega-diverse countries being the fourth nation in terms of species richness. The country presents almost all the climates of the planet, which, together with its rugged topography and complex geology, allows the virtual existence of all terrestrial ecosystems present in the world, concentrated in only two million square kilometers. On the other hand, two large biogeographic realms concur in the territory: the Neoarctic, which contributes a great representation of the species of the temperate zones of the world, and the Neotropics, which provides many elements of the tropical zone. As a result of the above, it is estimated that hundreds of thousands of species inhabit the country, with a very wide genetic variety, particularly evident in the case of cultivated species (Sarukhán et al., 2009). The country has an estimated 544 species of land and marine mammals, it is second in terms of mammalian species, as well as reptiles with 804 species, with between 300,000 and 425,000 estimated insect species and 23,522 known plant species. An estimated 32% of vertebrate fauna is endemic to the country and 52% is endemic to Mesoamerica. In Mexico around 30 million people live in rural areas and about 12 million belong to one of 86 ethnolinguistic indigenous groups that make up about 10% of the population (INEG, 2019).

#### Implementation of the CBD and the NP

Mexico has been a party of the CBD since it came into fruition (29 December 1993) and signed the NP on February 25, 2011. The ratification instrument was deposited on May 16, 2012, becoming the fifth nation to ratify it and the first mega-diverse country to do so. Although the protocol was signed nine years ago, it has not yet been effectively implemented due to legal lacunae.

The National Commission for the Knowledge and Use of Biodiversity (CONABIO) served as the National Focal Point for the Special Intergovernmental Committee of Open Composition of the NP, yet it ceased to function at the first meeting of the Protocol.

Now, the designated National Focal Point (NFP) is the General Direction of the Primary Sector and Renewable Natural Resources (DGSPRNR) of the Ministry of Environment and Natural Resources (SEMARNAT).

The Competent National Authority (CNA) is split into six government departments, delineated below.

The *National Service of seed Inspection and Certification,* Secretary of Agriculture is responsible for the coordination of policies, strategies, actions, and international agreements on access, conservation, and sustainable use of plant genetic resources (ABSCH, 2019). The *General Direction for Wildlife*, Secretary of Environment and Natural Resources (SEMARNAT) issues permits and other instruments for health, capture, collection, research, exploitation, possession, handling, reproduction, restocking, import, export, release, transfer of specimens, and derivatives of wildlife species (absch-cna-mx-238565-1 National/Federal Oct 22, 2017). The *General Coordination of Livestock*, SAGARPA issues guidelines for the granting of genealogical registration certificates and those related to the evaluation of the genetic value of the breeding stock used in the genetic improvement of livestock species. (absch-cna-mx-238023-1 National/Federal 10 Aug 2017). The *General Direction of Forest and Soil Management* (DGGFyS), Secretariat of Environment and Natural Resources (SEMARNAT) Is responsible for granting authorizations for the sustainable use, conservation, protection, and restoration of forest resources and the corresponding soils, for the collection and use of forest biological resources for scientific purposes. (absch-cna-mx-203872-2 National/Federal 08 Aug 2017). The *National Commission for the Development of Indigenous Peoples* (CDI) guides, coordinates, promotes, supports, encourages, monitors, and evaluates programs, projects, strategies, and public actions for the integral and sustainable development of indigenous peoples and communities (absch-cna-mx-203818-2 National/Federal 08 Aug 2017). The *National Commission of Natural Protected Areas* safeguards Natural Protected Areas (ANP), genetic diversity of wild species, as well as engaging in the preservation and sustainable use of species in some risk category (absch-cna-mx-238008-1 National/Federal 08 Aug 2017).

Furthermore, the Government of Mexico, as an administrative measure, created the inter-secretarial group for the implementation of the Protocol, which is made up of 22 Federal Government Departments and has defined the current access policy in Mexico and agreed the procedures for attention permits/access resolutions.

By the end of 2019, the country reports eight Internationally Recognized Certificates of Compliance (IRCC).

#### The NP and Basic Research Within México

Another gap in the NP is the lack of a clear definition of the “provider” and the “user.” The protocol refers to countries, or countries and foreign companies, but it does not define the roles and relationship between stakeholders belonging to the same country. This becomes especially important when dealing with researchers (universities) who work with genetic resources and the communities where the biological resource is located. The question is whether such cases need to be regulated by the NP or by other kinds of national regulation. In the case of Mexico, of the eight IRCC, five are for research purposes in the same country, one for commercial ones, and only two for commercial purposes involving a foreign country. In this case, only the last three IRCCs fit in the aim of the NP. In many cases (medicinal plants, phytocosmetics, biological pesticides, etc.) the initial research is done at national universities, which in the best-case scenario results in the publication of a paper. Clearly the NP does not cover such research, but the question is how such research should be governed if provider and user of information and resources are based in the same country where the genetic resources originated. Currently, there is no consensus on this.

#### SWOT Analysis and Core Future Action Points

Since 1992, with the creation of the CONABIO, México has been proactively working on the conservation and sustainable use of biodiversity. However, issues arose translating the regulatory framework into a national policy of ABS, and in general, there are major concerns in México that the country and its population will not benefit from granting access (**Table 4**). The NP is a national law (Ley Suprema de Toda la Unión) of the entire nation (United Mexican States). When it is incorporated into the Mexican legal system, its application and specific invocation in the national territory by the Mexican authorities is legally valid. As seen in the SWOT analysis, the NP is institutionally well developed and, legally, biodiversity is protected from activities, such as hunting, logging, fishing, and illegal trade of species affected by overexploitation. In the country there are restrictions on the commercialization of at-risk species at the national level, according to the Official Mexican Standard (NOM-059).

**Table 4:** SWOT analysis of access and benefit sharing—Case study Mexico.

Strengths	Weaknesses
• Mexico, as a megadiverse country, signed the NP in 2011. **(B)**	• Mexico lacks specific legal instruments that grant attributions to the government; a precise legal definition of what comprises a genetic resource is lacking. **(R)**
• There have been several ABS agreements; some of them have involved benefit-sharing for the local communities (indigenous groups) from the commercialization the biodiversity. **(R/I)**	• Further implementation of the NP is needed to legally define responsibilities of the competent national authorities regarding ABS. **(R)**
• The infrastructure is well-developed, including strong academic stakeholders and an active pharmaceutical industry. **(A/I)**	• Indigenous consultation, particularly Prior Informed Consent (PIC), is lacking. **(R)**
	• There is a loophole for those cases of genetic resources that are not specifically stated in current laws, for example, some microorganisms or aquatic species that are not in any category of risk, in compliance with NOM-059-SEMARNAT-2010 or that are of aquaculture or fishing interest. **(R)**
	• In many cases, research and development processes are carried out in a different country and not in the one the resource was obtained from; therefore, the exchange of information between "user" country and the provider of the genetic resources, to verify legal and legitimate access, remains a challenge. **(R)**
**Opportunities**	**Threats**
• Once the country has fully implemented, the national regulations based on the NP will be of great benefit especially for rural populations (around 30 M) or to those who speak indigenous languages (around 12 M), as well as helping the country in the preservation of biodiversity. **(R)**	• Major concerns about drug trafficking, corruption, and political instability result in a lack of willingness of international stakeholders to develop links with institutions and companies (like agricultural cooperatives) in México and specifically in regions of high risk. **(G)**
• For a country in which more of the half of the population lives in poverty, a legally binding and enforceable implementation of the NP could result in social and environmental benefits, especially for them. **(G)**	• High levels of poverty result in illicit exploitation of biodiversity including clandestine logging and illegal trade in biodiversity. **(G)**
• Stakeholders among the local industry could become more interested in the sustainable use of biodiversity. **(I)**	

The legal implementation of the fair and equitable sharing of benefits arising out of the utilization of genetic resources would be of benefit, but México lacks a specific legal instrument that grants attributions in the matter. Some government departments are limited when it comes to the implementation of the protocol, since the exact definitions that are comprised or the type of genetic resources is not included. This is of particular concern related to people that live in rural areas or who speak indigenous languages.

Lacking a specific legal instrument to implement the Protocol, it became necessary to carry out coordinated administrative actions based on laws and procedures already in place, as well as in compliance with the powers of CONABIO. Consequently, permits have been issued to users who have complied with the procedures by guaranteeing the rights of providers of genetic resources and associated traditional knowledge in each case. As in other countries, the regulation is ambiguous and some genetic resources remain unregulated simply because they are not specifically mentioned in the current laws; e.g. microorganisms or aquatic species that are not in any category of risk, in compliance with NOM-059-SEMARNAT-2010, or that are of aquaculture or fishing interest. There is also a need to update the internal regulations of each designated government department so that, at an operational level, the CNA has staff with specific powers to address the issues. México has not yet designated institutions as check points to meet the provisions of the Protocol. Similarly, a law would be required to link the indigenous consultation component with the provisions of the Protocol, particularly the PIC and the distinction between collections for research purposes versus commercial use. In general, the challenges relate, not to the legal framework as such, but to its specific implementation, which is not currently regulated.

México is still in the process of developing the relevant regulatory, administrative, and political measures required for a mutually agreeable access to genetic resources. A first core action would be the promulgation of these administrative and political measures in the corresponding legislation.

As of 2020, the legal instrument to regulate the utilization of traditional knowledge associated with genetic resources, in which measures relating to indigenous peoples and local communities will be adopted, is being developed. The main challenge is represented by reaching a consensus over each of the laws’ provisions; the areas of competence of the national authorities involved and the authorization management; and the promulgation and implementation of the legal instrument which would be generated by the institution in charge of this task.

In 2016, the country received financial support from the United Nations Development Programme to increase, in a participatory manner, the capacities of national authorities (SRE, SEMARNAT, SAGARPA, and SE) in México, as well as improving the legal and administrative framework in relation to genetic resources, associated traditional knowledge and benefit sharing, according to the institutional conditions for the implementation of the NP. The program ended in January 2020 and resulted in some institutional coordination and a series of training programs for federal administrative staff. In the future the priorities for action need to be:

- Developing mechanisms for the protection of traditional knowledge and capacity building in local and indigenous communities;
- Raising awareness among relevant actors about the conservation and sustainable use of biodiversity, genetic resources, and associated traditional knowledge.

### Panama

#### The Flora of Panamá and Its Uses

Panama is a biogeographic bridge between the flora and fauna of Central and South America. Panama’s territory has 10,444 known species of plants that represent 3.3% of the world’s diversity. Of these, 9,520 are vascular and 938 species are ferns and related groups; of the 924 non-vascular plants, 796 are species related to mosses. The endemic species of Panama amount to 1,300, of which 1,176 are plants. Panama has 21 times more plant species per km^2^ than Brazil (National Biodiversity Strategy of Panama, Ministry of Environment, 2018), ranking 11th in biodiversity per land area. These make Panama an attractive party for those seeking opportunities for discovery of active molecules.

Different species of flora in Panama are used for food, cultural, and medicinal use among different indigenous and other minority groups which according to the 2010 National Census represent 12.3% of Panama’s population (INEC/UNFPA, 2010). These include Afro-descendants and eight indigenous groups: Kuna, Ngäbe, Buglé, Teribe/Naso, Bokota, Emberá, Wounaan, and Bri Bri. There have been a number of efforts to document some of these uses, although few have focused on their commercial potential (Chízmar et al., 2009; Caballero-George and Gupta, 2011; Bermúdez and Ramos Chue, 2014; ANCON, 2017; Ross et al., 2019).

#### Implementation of the CBD and the NP

Panama’s National Assembly ratified the NP on October 12, 2012. Prior to its ratification, in 2005, a decree regulating Article 71 of Panama’s General Environmental Law (Law 41 of 1998) on the use and control of genetic resources was issued. However, this law did not help to promote collaboration and stakeholders requested its revision. In 2009, Executive Decree no 25 of April 29 enhanced the regulations on use, access, and control of genetic resources. The latter was reviewed and a new ABS Executive Decree was issued in 2018. Panama’s focal point for the NP is the Ministry of Environment which established SARGEB (Section for Access to Genetic and Biological Resources) to be in charge of norms, regulations, and controls. This section of the Ministry of Environment issues permits to applicants to conduct research with commercial and non-commercial objectives and is the contact with the CBD clearing house.

Molecules found in species under research in Panama have already been the subject of international ABS agreements. New active compounds have been identified, a repository of microorganisms has just been launched and several contracts with international companies have been signed (Biobanco, 2019).

#### SWOT Analysis and Core Future Action Points

Undoubtedly, Panama has taken important steps to put in practice the ABS contemplated in the NP (**Table 5**). Its large biodiversity, more than a decade of research on natural products with agreements in place with local communities, and a straightforward support of its government, place Panama in the forefront of the implementation of the ABS. From the perspective of a commercial use, the level of royalties is a realistic expectation and it is one of Panama’s key strengths from this perspective. More refinement needs to be done to fill gaps that would prevent delays in the interpretation of some sections of the recently-issued decree. What matters the most is that all the stakeholders are acting in good faith and willing to comply with the ABS to make Panama a model for the rest of the world.

**Table 5:** SWOT analysis of access and benefit sharing—Panama.

Strengths	Weaknesses
• Before ratifying the NP, Panama had already implemented ABS projects through interinstitutional and multi-stakeholder agreements. This led to Panama being the first country ever to receive funding under the CBD convention. **(R)**	• Working with traditional knowledge of indigenous groups is still a challenge. The lack of trust by indigenous people might hinder negotiations, to the point where external stakeholders could only look for opportunities outside indigenous territories. **(G)**
• Panama ratified the NP in 2012. An executive decree regulating access to genetic resources was issued in 2009 and reviewed in 2018, indicating a very high level of experience in translating the international agreements into national practice. **(R)**	• The sharing of benefits is not well understood by the general population. **(K)**
• Panama has a network of research institutions already collaborating with local environmental NGOs and the government in order to ensure that CBD guidelines are compatible with stakeholder requirements. **(A)**	• The traditional knowledge or rights of some minorities might be in conflict with the potential contribution to humanity of that knowledge. **(G)**
• The new ABS decree has established a level royalty for the Government of 1% of net sales, which is acceptable to the relevant industries. **(R)**	• The inventory of traditional knowledge is incomplete. Traditional knowledge is anecdotal and needs to be registered. **(A)**
	• The fact that the decree does not indicate the geographic or jurisdictional extension of the benefit rights of the traditional knowledge means that demands could arise from members of the same ethnic groups located outside of the collections site(s) of the traditional knowledge owners. **(R)**
**Opportunities**	**Threats**
• There is considerable commercial interest in working in Panama. **(I)**	• When governments have to negotiate MATs on behalf of both communities and industries, the former could perceive that the government is not protecting their right, resulting in internal conflicts within the country. **(R/G)**
• Local research organizations have experience in bioprospection studies. **(A)**	
• The well-established democracy of Panama and its stable economy give confidence to the industries interested in working with Panama with shared resources under ABS rules. **(R)**	
• There are reputable and ABS knowledgeable organizations other than the Government, the traditional knowledge owners, and the Industry that help to ease the lack of trust of the negotiation processes, especially prevalent within the indigenous groups. **(R)**	

#### Developing a Collaboration between Panama and an External Partner—A Case Study

The ABS process in Panama, and challenges encountered, can be illustrated with one practical example of a project. With the financial support of the German Agency for International Cooperation (GIZ) under a regional ABS project, ANCON (the National Association for the Conservation of Nature), an environmental NGO in Panama prepared a Catalogue of Native Species with commercial potential found in their largest private reserve (Panama’s largest privately owned reserve), which prompted the interest of a company in the UK. Thanks to the coordination efforts of GIZ, a Memorandum of Understanding was signed in 2018 between ANCON, a research institute (INDICASAT AIP), and Indigena Biodiversity Ltd., a UK facilitator specializing in ABS, to conduct research on the species highlighted in the catalogue. The parties quickly identified their roles and after a short negotiation process, they executed a MAT for a R&D project that included an additional research party based in the UK, as explained below.

An access application was filed involving a plant species which is a natural antimalarial, but with a poor therapeutic/toxicity ratio. The species’ samples could be collected through an application filed by ANCON; INDICASAT was then in charge of extracting and testing the material to be exported through Indigena Biodiversity. The UK-based third-party research partner was to investigate novel compounds, derivatives of the natural ones extracted from the plant samples, with improved antimalarial activity.

It is important to highlight that, in order to get to this point, it was critical to first establish an effective local partnership to seek common ground between the commercial entity in the United Kingdom and the two stakeholders in Panama. The parties initially differed on some issues, but through effective and thoughtful communication, collaborations were established. Contacts were also made with the CBD Focal Point in Panama, represented by the relevant government Ministry of Environment, MiAMBIENTE, Department of Biodiversity and Wildlife. The partnership that was developed implemented a series of core principles and mutually agreed terms. Some key observations were:

- Local research
  Access regulations require that some research be carried out in Panama. Local involvement is considered by Panama partners as part of the non-monetary benefits, including the opportunity for scientific publications. The project was able to comply with that requirement by having extraction and testing carried out locally. That modest contribution was acceptable to one UK research partner. However, a second UK company was not willing to delegate those steps, resulting in a lost opportunity for both parties.
- Access application
  The formal requirements of an access application in Panama are reasonably modest, although names of individual UK researchers are requested, as well as identification documents, including notarized copies of passports. This process, without a knowledgeable local partner, may deter a less committed user. The application took about 6 months to be granted.
  A permit for commercial research requires a MAT agreement to be in place. However, it is possible to initiate the process by obtaining a permit for non-commercial research in order to collect material; and then re-apply when terms have been negotiated, and there is some evidence of commercial potential. Although it might seem duplicative, and somewhat illogical, it is a pragmatic work-around for the common problem of MAT timing.
- Level of benefit-sharing
  At the time of the access application for this project, there was a benefit-sharing requirement of a minimum 1% royalty of net sales among the parties involved. With the revised ABS decree of 2018, a 1% royalty of net sales for the government was established, to avoid a long negotiation process. An overall percentage royalty has been negotiated, but the distribution of that figure between the stakeholders has not been finalized.
- Traditional Knowledge
  The species that is the basis for this project is widely used throughout Panama, so that any traditional knowledge is nationally disseminated and not attributable to any indigenous community. Whether such disseminated traditional knowledge gives rise to a benefit-sharing obligation under CBD/NP is an open question. However, the parties in this project wish to follow the broad principles of the CBD, so plan to put a proportion of any benefit into a community fund.
- Intellectual Property
  There was a broad consensus about IP issues. On a practical level, however, the issue was more related to pressure to share IP ownership derived from the extraction and testing activities. However, this is all part of the negotiation process. It is well established that mere testing does not constitute an inventive contribution and that was eventually accepted.
- Mutually Agreed Terms
  Once the good working relationship was established, although there were issues to resolve, from a commercial perspective, obtaining a MAT agreement was no more difficult than any other negotiation. The partners were free to consider and debate all the issues. Terms were successfully agreed within 6 months.
- Prior Informed Consent
  A good partnership of trust also facilitated the granting of a PIC. Given that the landowner promotes the sustainable use of Panama’s natural capital through biocommerce and bioprospection projects, as activities that could bring significant benefits to communities and to biodiversity conservation, it was straightforward to draft and agree a simple PIC document. One month from start to finish. As stated above, this project did not involve associations with any specific indigenous community, but the local partnerships that are in place provide the basis for future community collaborations.

ABS regulations in Panama still need some enhancements to attract foreign industry, but the present system is workable for motivated users, as exemplified in this case study.

### Peru

#### The Peruvian Flora and Its Uses

Peru harbors an estimated 78 of the 107 eco-regions of the world, having 17,143 taxa of spermatophytes in 2,485 genera and 224 families; the flora of the country represents 10% of the world’s total, of which 30% is endemic (Bussmann and Sharon, 2014). Peru is the fifth country in the world in number of plant species with known properties utilized by the population (4,400 species) with 1,400 species described as medicinal (Brack Egg, 2004). Peru’s indigenous groups make up from 26-30% of its total population depending on the quoted source (Brack Egg, 2004), with 95.8% of these located in the Andean region and 3.3% in the Amazon. In the latter region alone, there are more than 65 ethnic groups classified into 16 language families. Modernization results in an enormous loss of traditional knowledge relevant to the indigenous peoples and of great value to the science and technology of Peru (Bussmann, 2013). In all Peruvian indigenous groups, plant knowledge is of extreme practical value; and also reinforces national identity and values, which are being lost in the complementary processes of modernization and globalization. Medicinal plant commerce in Peru is a major economic resource with 510 medicinal plants and 974 remedies of mixtures recorded (Bussmann and Sharon, 2007; Bussmann and Sharon, 2014). Peruvian national sales of natural products derived from medicinal and aromatic plants are steadily growing and now exceed $400 million per year (UNCTAD, 2018). This offers huge opportunities for the country that could largely benefit from an efficient and effective implementation of an ABS scheme.

#### Implementation of the CBD and the NP

Peru ratified the CBD on 29 December 1993 and has been party to the NP since its creation in 2014. The legal framework for its application in the country is built upon the following laws: a) Law no. 27811 on the Regime for the protection of collective knowledge of indigenous peoples related to biological resources; b) S.D. N° 003-2009-MINAM Supreme Decree that approves the Regulation R.M. no. 087-2008-MINAM for Access to Genetic Resources; c) Decision no. 391 of Andean Community establishing the Common Regime on Access to Genetic Resources; d) Decision N° 486 of Andean Community Establishing the Common Industrial Property Regime; and e) Law N° 28216 on the protection of access to Peruvian biological diversity and the collective knowledge of indigenous peoples.

Peru’s ABS National Focal Point (NFP) is the Ministry of Environment (MINAM) that operates through five Competent National Authorities (CNA). One of them is MINAM itself, while the others are the National Forest and Wildlife Service (SERFOR), the National Institute of Agricultural Innovation (INIA), the Ministry of Production (PRODUCE), and the National Institute for the Defence of Free Competition and the Protection of Intellectual Property (INDECOPI).

Peru’s checkpoints are The Directorate of Inventions and New Technologies (DIN) of INDECOPI, and “The National Commission Against Biopiracy” (CNBio) that exercises control over illegal access to genetic resources and associated traditional knowledge.

So far, Peru has not issued any ABS contract for commercial purposes—though it has issued sixteen Internationally Recognized Certificates of Compliance (IRCCs) for non-commercial research. To date there are also three applications submitted to INIA currently under revision that are expected to lead to new IRCCs in the next few months (MINAM, 2019).

### SWOT Analysis and Core Future Action Points

As a signatory to the NP with a few signed contracts and being well recognized internationally for its achievements in fighting biopiracy, Peru has a strong basis for collaboration, even though the enforcement of Peruvian rights, for example relating to the use of dragon’s blood (*Croton lechleri* Müll.Arg.), maca (*Lepidium meyenii* Walp.), and sacha inchi (*Plukenetia volubilis* L.), remains unresolved. As in other countries, core concerns relate to the lack of information about the potential and limitations of ABS-projects. Peru is struggling to update its procedures and regulations in order to provide a timely response to researchers and other interested parties that request formal access to genetic resources (Silvestri, 2016; Friso et al., 2020) and the financial expectations are seen as being unrealistic. So regulatory uncertainty is a core concern (**Table 6**).

**Table 6:** SWOT analysis of access and benefit sharing—Case study Perú.

Strengths	Weaknesses
• The Peruvian Amazon holds great biodiversity, and over 1000 medicinal and food plants with commercial potential have been recorded. **(B)**	• There is generally poor information about the legal framework for access to genetic resources and associated traditional knowledge, and indigenous and local communities have no control over an illegal access by national or foreign users. **(K)**
• As a signatory to the NP with a few signed contracts, Peru is ahead of other signatory countries. So far, the application of the NP has generated both monetary benefits and non-monetary benefits, the latter considered greater than the former. **(R)**	• The ambiguity of Peruvian regulations generates concerns for many stakeholders. **(R)**
• Peru's CNBio is recognized internationally for its achievements in fighting illegal extraction of biodiversity. **(R)**	• Peru is struggling to update its procedures and regulations in order to provide a timely response to researchers and other interested parties that request formal access to genetic resources. **(R)**
• The National Council of Science, Technology and Technological Innovation (CONCYTEC), the General Directorate of Environmental Health (DIGESA), and the Directorate General of Medicines, Supplies and Drugs (DIGEMID) are all competent authorities potentially able to verify and monitor the stages of research, development, and commercialization following an ABS agreement. NGOs and international cooperation agencies are well established to complement this effort, helping to balance the power relations between North and South in the field of intellectual property and distribution of benefits. **(R)**	• The mandatory monetary benefits (5% to provider organization and 10% of gross sales to the national Fund for the Development of Indigenous Peoples) are considered too high from a commercial perspective. **(R)**
	• There is a relatively weak local industry interested in the use of biodiversity. **(I)**
**Opportunities**	**Threats**
• Peru could participate much more actively in the research and development processes relating to patenting of plants and biological materials in collaboration with foreign partners from more developed countries. **(A/I)**	• Gaps between the regular access that passes through the ABS system and ones outside of it pose major risks for R&D. **(R)**
• Existing examples of products under development offer the chance to demonstrate how collaborative projects can be developed. **(I)**	• Peru's neighbors such as Colombia and Brazil have not signed up to the NP and international companies, and researchers can access there the very same genetic resources with the associated traditional knowledge avoiding the complexity of the Peruvian procedure. **(R/G)**
	• Inflexible attitude of regulators can crush well-intended development initiatives (like Biotrade). **(R)**

Starting in April 2018, an internationally funded training and implementation project is being carried out to put into practice the NP in Peru, addressing many of the previously reported weaknesses and threats. Additionally, a national communication strategy is being implemented to disseminate among indigenous peoples the norms and mechanisms on protection of traditional knowledge and provide them with the necessary know-how to become involved in access negotiation.

In addition, MINAM is proposing a new regulation for the access to genetic resources (MINAM, 2019). Among the main changes of this new law, whose overall objective is to facilitate research and mark a clear difference between access for non-commercial and commercial purposes, there is an additional exemption for basic non-commercial research related to the identification, delimitation and classification of species for taxonomic, systematic, and phytogeographic purposes. The new law also aims at giving greater importance to the National Support Institutions, specifying their role, rights and obligations when assisting foreign applicants in access activities (Friso et al., 2020). Changing the law, however, is only one step toward the correct implementation of NP in Peru; even more important would be the training of the public officials involved in this area, to facilitate and speed up the overall ABS process for researchers or private companies interested in passing through this mandatory procedure. A training program delivered by international experts coming from those countries where the NP has been better implemented could be therefore recommendable.

## Overall Conclusions

Both the CBD and the NP are crucial elements for developing equitable partnerships among countries with a focus on the potential benefits of products resulting from basic and applied research on such resources. The nearly universal adoption of the CBD and the widespread acceptance of the NP have changed the frameworks of collaboration and provide a much better basis for collaborations aiming at sustainable and equitable development of resources. Using biodiversity in the development of high value products remains a very promising and, at the same time, contentious area. It is a fast developing area of discussion also linked to technological changes. Thus, we need to be aware that genetic resources can now be accessed as digital sequence information (DSI) rather than as biological material (Convention on Biological Diversity, 2019b). Here we reiterate, that any ABS framework will need to take into account the consensus view that: “Benefit-sharing arrangements for commercial and non-commercial use of DSI should reflect the same or similar benefit sharing obligations as those attached to biological materials” (Convention on Biological Diversity, 2019b).

Here we draw upon current observations from six provider countries in Latin America to identify some of the hurdles; and provide recommendations for a way forward.

In this comparative analysis, some obvious outcomes are relevant. In general, all countries have a high or very high biological diversity and most also have a considerable cultural diversity, as seen in the existence of indigenous groups mostly occupying biodiverse-rich territories. The hurdles to creating opportunities from that biological and cultural diversity fall into the following categories.

1. NP implementation
  The first challenge, common to all the case studies, is that the political framework and the implementation of NP policies can in itself produce contentious problems. Often, there seem to be limited or no benefits from implementing the NP. Clearly, there are far fewer parties that have ratified the NP as compared to the CBD. The NP demands the creation of a complex regulatory framework to oversee its implementation, which can cause both internal social conflict and deter international investment, subsequently defeating the objective of the CBD. Since many provider countries have a rich biodiversity, a user from another country can choose between different countries. Consequently, from the perspective of competing macroeconomic interests, implementation of the NP can in fact be detrimental, since countries which offer “easier” opportunities for access may benefit commercially. Yet, at the same time, the treaties offer an opportunity to implement new, more equitable policies at national level. It calls for processes which empower participants and provide platforms for developing a mutually agreed national strategy. Many countries, including Panama and Guatemala, are making efforts to take on board some of these policies. Although beyond the scope of the present comparative analysis, the regulatory framework in question needs to be integrated into a broader set of government policies and activities, including the need for protection, redistribution, and access to land for indigenous people and an equitable sharing of potential economic benefits.
2. Expectations of benefits
  The second hurdle observed in many of the countries in our analysis is an unrealistic expectation of the level of benefit. It is the interests of biodiversity-rich countries to stimulate ease of use of the CBD to generate benefits from their genetic resources which is equitable to all stakeholders. The benefits may be monetary, as well as technological development, and often access to new medicines relevant to local populations. However, if expectations are seen as unrealistic, potential users will be deterred. To encourage a successful application of the CBD and NP, users and providers of genetic resources need to understand each other’s perspective. The inclusion of non-monetary benefits in bilateral agreements could help to establish trust between the provider community and the user party. Benefits, such as exchange of knowledge and one-off or continued support for local projects/initiatives, could be provided independent of commercial outcomes. This could encourage long lasting collaborations as a benefit delivered at a local level.
3. Indigenous communities and traditional knowledge
  Since biodiversity and the land are tightly connected with people’s livelihoods, engagement with their environment could provide an impetus for local development, provided that a new basis for collaboration between stakeholders is developed and that this is communicated in a transparent way. There is a risk that the NP creates more endogenous civil unrest than it provides consensus among indigenous peoples and within countries. The problem can be more exaggerated in countries where there is less political representation of indigenous peoples. The experience of hundreds of years of exploitative relationships cannot be overcome through international treaties and indigenous groups and other national stakeholders often see such international treaties as a neo-colonial mechanism.

### Recommendations

Drawing together the issues we have identified and based on the fundamental ethical and legal principles as outlined in the treaties, key requirements for a successful framework to implement CBD and NP include:

- A country’s ABS regulations should provide a framework that is attractive to foreign basic and applied research as well as commercial entities including legal clarity;
- Realistic expectations of the level of benefit available from commercialization;
- The need to incentivize partnerships with industry in user countries and to simplify procedures and allow freedom to negotiate;
- A more systematic approach ascertaining that non-commercial benefits to communities and regions are implemented providing direct local benefits;
- The management of ABS-related perceptions and the establishment of functional partnerships between stakeholders, in particular indigenous people and communities in provider countries;
- Participatory approaches including the recognition of fundamental rights of the often-marginalized people who have been the custodians of the local biodiversity and the associated knowledge for centuries;
- Particular exemptions will be needed for the assessment of the safety of herbal medicines, especially in the context of local uses of toxic species (Michl et al., 2014);
- Professional development of stakeholders in the principles of the CBD and NP and the respective implementation at a national level.

The effective formulation of a legal regime of access to genetic resources requires the participation of a large number of interest groups and experts. The discussion on how to regulate genetic resources should be carried out through a national planning process, as required by Article 6 of the CBD, and it should be based on a mechanism which results from a broad consensus on the strategic national goals and how to achieve them. Government entities from different sectors must participate in the process, as well as representatives of the scientific community and the private sector (for example, pharmaceutical and agricultural companies), indigenous people/communities and NGOs. A core concern relates to the challenges associated with the consultation processes with indigenous groups, an unresolved problem in several of the countries presented here, including Chile, Guatemala, México, and Colombia.

Such a process also allows the establishment of broader objectives and national policies, while facilitating the evaluation of existing institutions, laws, and policies. Since the regulation of access to genetic resources is a new area of legislation, few countries have the necessary institutions and resources for its implementation. Developing this capacity requires a long-term process and, therefore, it is vital to start it as soon as possible. However, considering the elements mentioned above, it is clear that there are tensions between the urgent need to take action and the complexity of the process. With the analysis of the implementation of ABS mechanisms in these six countries, we were able to show that—in order to secure benefits from the NP, most importantly—a national strategy is needed that facilities a better understanding of the treaties and the resulting opportunities for a more equitable development.

## Author Contributions

AA-C, MB-G, JE, FF, FG-C, MP, RS, and CS contributed with their experience on the implementation of the CBD in the respective countries, AH provide insight into industrial and IP aspects (as well as on the situation in Panamá and Guatemala), CM on the academic perspective of the implementation of the treaties, MHi assessed conservation related aspects. FS contributed to the section on Guatemala and the overall writing of the manuscript. MHe conceived the idea, organised the initial symposium and lead the gathering of data on the situation in the respective countries, also writing introduction and conclusions as well as overseeing the development of the entire MS. All authors intensively discussed the data, the overarching concept and the entire manuscript.

## Funding

MB-G’s and FS’s positions are co-funded by the UK’s Department of Food, Environment and Rural Affairs under its Darwin Initiative (26-005: Green Health: improving indigenous participation through the CBD’s ABS mechanisms). This project also includes funding to Indigena Biodiversity Ltd. In addition, FS’s position is co-funded by a grant of the Velux Stiftung, Switzerland. Additional funding by UCL’s Global Engagement Office for a two-day meeting on the implementation of the NP in the six countries is gratefully acknowledged. The German Cooperation Agency (GIZ), through a regional ABS project, supported stakeholder collaboration and the review of Panama’s regulation on access to genetic and biological resources. CS was partly funded by the National System of Investigation (SNI) from Panama and by a Global Environment Facility grant (GEF/PNUD 81860). MB-G position has been co-funded by the SNSF R4D Grant 177385. MB-G was co-funded by the SNSF R4D Grant 177385. AA-C was partly funded by the project DGAPA, PAPIITIN 226719. JE gratefully acknowledges funding from CONICYT(PAI/ACADEMIA N°79160109).

## Conflict of Interest

AH is employed by Indigena Biodiversity Limited.

The remaining authors declare that the research was conducted in the absence of any commercial or financial relationships that could be construed as a potential conflict of interest.

## Abbreviations

ABS, access and benefit sharing; CBD, Convention on Biological Diversity; CAN, Competent National Authority; CONABIO, National Commission for the Knowledge and Use of Biodiversity; CONAP, National Commission for Protected Areas (Consejo Nacional de Areas Protegidas (Guatemala); DSI, digital sequence information; IRCC, Internationally Recognized Certificate of Compliance; MAT, Mutually Agreed Terms; MTA, Material Transfer Agreement; NFP, National Focal Point; NP, Nagoya Protocol; PIC, Prior Informed Consent.
